# Development and first evaluation of a novel multiplex real-time PCR on whole blood samples for rapid pathogen identification in critically ill patients with sepsis

**DOI:** 10.1007/s10096-018-3255-1

**Published:** 2018-04-26

**Authors:** Kirsten van de Groep, Martine P. Bos, Paul H. M. Savelkoul, Anna Rubenjan, Christel Gazenbeek, Willem J. G. Melchers, Tom van der Poll, Nicole P. Juffermans, David S. Y. Ong, Marc J. M. Bonten, Olaf L. Cremer, on behalf of the MARS consortium

**Affiliations:** 1 Department of Epidemiology, Julius Center for Health Sciences and Primary Care, University Medical Center Utrecht, Utrecht University, Utrecht, the Netherlands; 2 Department of Intensive Care Medicine, University Medical Center Utrecht, Utrecht University, Room F06.149, P.O. Box 85500, 3508 GA Utrecht, The Netherlands; 3 Microbiome, Amsterdam, the Netherlands; 4 Department of Medical Microbiology & Infection Control, VU University Medical Center, Amsterdam, the Netherlands; 5 Department of Medical Microbiology, Maastricht University Medical Center, Maastricht, the Netherlands; 6 Department of Medical Microbiology, Radboud University Medical Center, Nijmegen, the Netherlands; 7 Center of Experimental and Molecular Medicine, Academic Medical Center, University of Amsterdam, Amsterdam, the Netherlands; 8 Division of Infectious Diseases, Academic Medical Center, University of Amsterdam, Amsterdam, the Netherlands; 9 Department of Intensive Care, Academic Medical Center, University of Amsterdam, Amsterdam, the Netherlands; 10 Department of Medical Microbiology, University Medical Center Utrecht, Utrecht University, Utrecht, the Netherlands

**Keywords:** Bloodstream infection, Sepsis, Intensive care, Diagnosis, Multiplex real-time PCR

## Abstract

Molecular tests may enable early adjustment of antimicrobial therapy and be complementary to blood culture (BC) which has imperfect sensitivity in critically ill patients. We evaluated a novel multiplex real-time PCR assay to diagnose bloodstream pathogens directly in whole blood samples (BSI-PCR). BSI-PCR included 11 species- and four genus-specific PCRs, a molecular Gram-stain PCR, and two antibiotic resistance markers. We collected 5 mL blood from critically ill patients simultaneously with clinically indicated BC. Microbial DNA was isolated using the Polaris method followed by automated DNA extraction. Sensitivity and specificity were calculated using BC as reference. BSI-PCR was evaluated in 347 BC-positive samples (representing up to 50 instances of each pathogen covered by the test) and 200 BC-negative samples. Bacterial species-specific PCR sensitivities ranged from 65 to 100%. Sensitivity was 26% for the Gram-positive PCR, 32% for the Gram-negative PCR, and ranged 0 to 7% for yeast PCRs. Yeast detection was improved to 40% in a smaller set-up. There was no overall association between BSI-PCR sensitivity and time-to-positivity of BC (which was highly variable), yet Ct-values were lower for true-positive versus false-positive PCR results. False-positive results were observed in 84 (4%) of the 2200 species-specific PCRs in 200 culture-negative samples, and ranged from 0 to 6% for generic PCRs. Sensitivity of BSI-PCR was promising for individual bacterial pathogens, but still insufficient for yeasts and generic PCRs. Further development of BSI-PCR will focus on improving sensitivity by increasing input volumes and on subsequent implementation as a bedside test.

**Electronic supplementary material:**

The online version of this article (10.1007/s10096-018-3255-1) contains supplementary material, which is available to authorized users.

## Introduction

Rapid detection and identification of a causative pathogen is essential in the treatment of critically ill patients with blood stream infection (BSI), since timely initiation of adequate antibiotic treatment is associated with decreased morbidity, mortality, and possibly reduced healthcare costs [1–5]. Furthermore, faster pathogen identification may limit the inappropriate use of antimicrobial drugs and consequently may reduce development of antibiotic resistance [1]. Conventional culture of inoculated blood samples, termed blood culture (BC), is currently considered the “gold standard” for diagnosing BSI. However, its diagnostic accuracy may be hampered by concomitant antibiotic treatment, low levels of circulating bacteria, and poor sensitivity for slow growing, intracellular, and fastidious microorganisms [2, 6, 7]. In addition, BC requires between 24 and 72 h to yield final results [7].

Polymerase chain reaction (PCR)-based assays performed directly on whole blood may complement BC, by yielding faster results and improving pathogen detection [7]. In contrast to molecular tests performed on bacterial isolates, these assays are independent of time-consuming culture methods. However, highly variable diagnostic performance has been reported, both for the most widely studied LightCycler Septi*Fast* (LSF) test (Roche Diagnostics GmbH, Mannheim, Germany) [8, 9], as well as for various other PCR-based detection technologies [10–12]. In an open-label, cluster-randomized, interventional crossover trial, the use of LSF test in septic patients increased the proportion of patients with a microbiological diagnosis, but failed to reduce 7-day mortality [13]. Consequently, the utility of these tests during routine clinical practice remains unclear as molecular techniques continue to be refined [2, 14]. For example, the efficacy of the LSF test may be impeded by lengthy hands-on time (ranging 5–7 h) and its sensitivity may be compromised by the use of relatively low blood volumes for analysis (i.e., an equivalent of 0.17 mL of blood per PCR tested).

We developed a novel multiplex real-time PCR assay (which will further be referred to as BSI-PCR) for detecting microbial DNA directly in relatively large-volume whole blood samples (5 mL) of patients with presumed infection, targeting the bacteria, fungi, and resistance markers considered most relevant for intensive care unit (ICU) populations [15, 16]. In the near future, this assay will be implemented in a point-of-care platform requiring only very little hands-on time and yielding results within 3 h. This will enable physicians to confirm the presence of infection and adjust the empirical antimicrobial regimen early, while BCs are still being processed. In this manuscript, we describe essential steps taken during BSI-PCR development so far, and report a first systematic evaluation of test characteristics in a large selection of clinical blood samples obtained from ICU patients with suspected infection. Of note, this study did not (yet) aim to evaluate the clinical impact of the new multiplex PCR on diagnostic reasoning and merely focuses on sensitivity of individual PCRs rather than predictive values or specificity of BSI-PCR as a whole.

## Materials and methods

### BSI-PCR assay design

For detection of pathogens at species level, we designed novel PCRs for *Escherichia (E.) coli*, *Enterococcus (E.) faecium*, *E. faecalis*, *Acinetobacter (A.) baumannii*, and *Staphylococcus (S.) aureus*, because previously reported assays were not sufficiently specific or inclusive in silico analyses (Table 1). We aimed to find multi-copy targets on the genome to increase sensitivity by using the PCR MultiMPrimer3 tool [25]. This resulted in 2-copy targets for both *E. coli* and *S. aureus*. For *E. faecalis* a 3–4 copy target was found in the sequence encoding RNA previously designated as non-coding [26]. For *A. baumannii*, a 6-copy target was identified in the 23S rRNA gene. The Insignia tool [27] provided a single copy species-specific sequence for *E. faecalis.* We also included a broad PCR that detects less prevalent pathogens; this PCR which we refer to as molecular Gram stain, discriminates clinically relevant Gram-negatives from Gram-positives [19]. Furthermore, detection of *Candida* species, *Aspergillus* and the resistance markers *mecA* and CTX-M1,9 were added to the test. Presence of CNS was inferred from positivity in the *mecA* and/or Staphylococcus genus PCR and negativity in the *S. aureus* PCR.

**Table 1:** PCR targets used in the BSI-PCR assay

Pathogen/resistance marker	Target	Target copy number per cell	Reference
*A. baumannii*	23S rDNA	6	This study
3Candida ^a, b^	ITS2	Variable	This study, [17]
*C. albicans*			
*C. glabrata*			
*C. krusei*			
CTX-M1,9	blaCTX-M1,9	Variable	[18]
*E. coli*	gadA gadB	2	This study
*E. faecalis*	ncRNA Ref12A	3–4	This study
*E. faecium*	Hypothetical ORF	1	This study
Enterococcus genus	23S rDNA	4–6	This study
Gram-neg/pos ^c^	16S rDNA	1–8	Adapted from [19]
*Klebsiella* species	rhaA-rhaD intergenic region	1	[20]
*mecA*	*mecA*	1	[21]
Pan-Aspergillus	ITS2	Variable	[20, 22]
*P. aeruginosa*	phzE	2	[20]
*S. aureus*	hsdM	2	This study
*S. pneumoniae*	comX	2	[23]
*Staphylococcus* genus	tuf	1	Adapted from [20]
IAC	Artificial	Not applicable	[24]

*IAC* internal amplification control

^a^3Candida PCR detects *C. albicans*, *C. tropicalis*, and *C. parapsilosis*

^b^All *Candida* species are detected with the same primer set combined with species-specific probes

^c^Gram-positive/Gram-negative detection is based on one primer pair with two different probes

Primers and probes were designed by MultiMPrimer3 or PrimerExpress (ABI) and their genomic targets are provided in Table 1. PCR amplicons were regularly tested for specificity and coverage in silico, as during the course of this study continuously new genome sequences became available. An internal control PCR was developed for each multiplex reaction by constructing a plasmid containing two primer sequences of the multiplex with an artificial probe sequence in between. Primers comprised of BSI-PCR target sequences and sequences complementary to pICwhi2 were used to generate an amplicon using pICwhi2 as template [24]. The amplicon was cloned into pGEM-T-easy, transformed into *E. coli* DH5a and then sequenced. Purified plasmid was added to each multiplex PCR mix in a concentration of approximately 150 plasmid copies per PCR reaction. The complete lay-out of the BSI-PCR assay is shown in Table 2.

**Table 2:** Composition and LightCycler settings of the BSI-PCR assay

Channel settings (Ex-Em)	Probe label	Multiplex 1	Multiplex 2	Multiplex 3	Multiplex 4
440–488 nm	Atto425	Pan-*Aspergillus*	*E. coli*	*S. aureus*	*Staphylococcus* genus
465–510 nm	FAM	Gram-positive	*E. faecium*	*E. faecalis*	*Enterococcus* genus
498–580 nm	HEX/Yakima Yellow	Gram-negative	*A. baumannii*	*P. aeruginosa*	*Klebsiella* species
533–610 nm	CalFluor610	3*Candida* ^a^	–	*C. krusei*	*C. albicans*
533–640 nm	CalFluor635	*C. glabrata*	*mecA*	*S. pneumoniae*	CTX-M1,9
618–660 nm	Atto647N	IAC	IAC	IAC	IAC

All PCR reactions were performed on a LightCycler 480 II (Roche Diagnostics).

*IAC* internal amplification control, *Ex* excitation wavelength setting, *Em* emission wavelength setting

^a^3*Candida* PCR detects *C. albicans, C. tropicalis C. parapsilosis*

PCR reactions were performed on a LightCycler 480 II (Roche Diagnostics). Reaction mixtures contained 12.5 μL SensiMix II (Bioline) plus 2.5 μL primers and probes (Biolegio and Biosearch). For each sample, 10 μL DNA, representing 0.71 mL of blood, was added per PCR reaction. Used cycling conditions were 10 min of 95 °C followed by 45 cycles of 95 °C for 15 s and 60 °C for 1 min. Results were analyzed with the LightCycler software version 1.5.0 using Color Compensation to correct for fluorescence bleed-through. Primer and probes mixes were prepared in bulk and tested with positive and negative controls in a separate experiment before running the samples.

### DNA extraction and isolation

DNA isolation from pure cultures was done as previously described [20]. For pathogen DNA isolation from blood, blood samples were processed following the 5 mL manual Polaris protocol [28]. This method ensures chemical lysis of human cells and degradation of free DNA before isolation and chemical lysis of pathogens. The resulting pathogen lysates of 0.22 mL were supplemented with 1 mL of EasyMag lysis buffer (Biomerieux) to preserve DNA, and stored at − 80 °C till final analysis. After thawing, the samples were added to EasyMag containers holding 1 mL of AL buffer (Qiagen), where after DNA was isolated using the specific A protocol in an elution volume of 70 μL on an automated EasyMag device. For each run of 23 samples an isolation control, containing only EasyMag reagents, was taken along. Resulting DNA was stored at − 80 °C.

### Spiking experiments

Microorganisms were grown to exponential phase in Luria Broth, diluted in Luria Broth based on optical density measurements, and spiked in 5 mL of blood from healthy volunteers to yield loads ranging from 1500 to 1.5 organisms per milliliter of blood. Suspensions were plated to verify the loadings. Spiked blood was processed as described above, except for the − 80 °C freezing step.

### Collection of clinical blood specimens for PCR testing

Clinical blood samples were prospectively collected as part of the Molecular Diagnosis and Risk Stratification of Sepsis (MARS) initiative, a prospective cohort study in two tertiary mixed medical-surgical ICUs in the Netherlands [29]. Ethical approval for the study was provided by the Medical Ethics Committee of the University Medical Center Utrecht (UMCU), including an opt-out consent method (IRB No. 10-056C). From January 2012 till June 2014 blood specimens (5 mL) for BSI-PCR evaluation were drawn simultaneously whenever blood cultures were taken on clinical indications (i.e., at the discretion of the attending physician) by using the same catheter hub or venipuncture site for collecting an EDTA blood tube. Samples were anonymized for both patient characteristics and corresponding culture results by using coded identifiers. Subsequently, blood samples were stored at 4 °C for a maximum of 3 days before further processing following the protocol described above. All BCs were processed according to routine clinical protocols in the microbiology laboratories of both hospitals.

### Clinical sample selection

To study both PCR-specific and the overall BSI-PCR performance across a wide spectrum of relevant pathogens, we selected blood samples based on the results of the pairwise collected BC. To exclude potentially contaminated BCs, some predefined criteria were applied for Enterococci and CNS [30]. For these species, we excluded all samples yielding polymicrobial growth as well as samples associated with another BC taken on the same day that remained either negative or yielded any other pathogen. Similarly, we took measures to reduce the risk that the 200 negative control samples were in fact resulting from a false negative BC. To this end, we selected only specimens of patients with no positive BC on the same day, and excluded specimens collected on the first day of post-surgical ICU-admissions. Among these, 50% of the negative samples were derived from patients receiving no systemic antibiotic or antifungal therapy on the day of sampling or during the 2 days prior to that. For each PCR-target, a maximum of 50 samples containing at least 4.5 mL of blood were selected, preferably from different patients. In case of a poly-microbial BC result, each isolate was assessed separately for eligibility. PCRs for antibiotic resistance markers were not evaluated in the clinical samples, because of a low prevalence of MRSA and ESBL-positive bacteremia in the Netherlands [31, 32].

### Diagnostic evaluation of BSI-PCR

Technicians, who performed all BSI-PCR assays and reported the results, were blinded for corresponding culture results and clinical information. Sensitivity and specificity with exact confidence intervals were calculated for each PCR separately by using BC as the reference test. For determining specificity, each individual PCR was evaluated against the same set of 200 negative BC. In contrast, overall sensitivity and specificity of specific pathogen detection by BSI-PCR were evaluated by combining the results of all 11 species-specific PCRs contained in the multiplex set-up. Thus, each single blood sample contributed 11 separate results to this analysis. To assess whether BSI-PCR detection of pathogens was dependent on pathogen blood load, we performed multiple comparisons between samples yielding true positive versus false negative results. All comparisons between groups were made using Mann-Whitney *U* tests or Pearson’s chi-squared test, as appropriate. All analyses were performed using SAS Enterprise Guide 7.1 (SAS Institute, Cary, NC) and figures were made using GraphPad Prism version 7.02 (GraphPad Software, La Jolla California).

## Results

### BSI-PCR development

In order to obtain broad coverage of relevant pathogens, we designed the BSI-PCR assay to include both species- and genus-specific PCRs, as well as generic PCRs discriminating a broad panel of Gram-negatives versus Gram-positives (further referred to as the molecular Gram stain; Table 2). The latter PCRs were added in order to cover rare pathogens not included in specific PCRs. Furthermore, we included PCRs targeting the resistance markers *mecA* (present in methicillin-resistant *Staphylococci*) and CTX-M1,9 (present in extended spectrum β-lactamase producers). All PCRs were first tested in a monoplex setting and found to be highly specific across a broad panel of clinically relevant microorganisms (listed in Table S1). Nineteen monoplex assays were then combined into 4 multiplex PCRs, each containing a tailored internal amplification control (Table 2). The analytical sensitivities of the mono- versus multiplex PCRs were highly comparable when using DNA derived from spiked blood samples (Table 3). Furthermore, most PCRs did not generate false-positive signals in over 100 isolation controls and negative control blood samples analyzed during test development, except for the molecular Gram stain and, occasionally, the *A. baumannii* PCR. These false-positive signals probably originated from chemicals and consumables used during these tests, which are known to be frequently contaminated with environmental bacterial DNA [33]. Based on these first observations, negative cut-off Ct-values were set at 35 cycles for the *A. baumannii* and the Gram-positive PCR, and at 33 cycles for the Gram-negative PCR.

**Table 3:** Analytical sensitivities of monoplex and multiplex PCR for detection of pathogens isolated from spiked blood samples

PCR	Detection format	Number of genomes per PCR ^a^					
		1000	100	50	10	1	0
Gram-positive	Monoplex	23.5	27	28.1	30.6	33.2	36.6
	Multiplex 1	25.5	28.6	29.8	31.2	32.9	33.2
Gram-negative	Monoplex	24.0	27.5	28.6	30.9	33.5	33.7
	Multiplex 1	26	29.1	30.5	31.9	33	36.9
3Candida	Monoplex	27.7	30.8	32.2	34.1	Neg	Neg
	Multiplex 1	26.6	29.6	31.1	33.0	Neg	Neg
*C. glabrata*	Monoplex	28.2	31.6	33.5	34.5	37.8	Neg
	Multiplex 1	26.8	30.3	31.3	32.9	Neg	Neg
*E. coli*	Monoplex	27.8	31.0	32.1	34.2	38.8	Neg
	Multiplex 2	27.5	30.8	31.8	34.1	36.4	Neg
*E. faecium*	Monoplex	30.9	33.8	35.0	37.2	40.0	Neg
	Multiplex 2	29.9	32.9	34.3	36.2	39.6	Neg
*A. baumannii*	Monoplex	26.7	29.5	31.1	32.7	37.0	Neg
	Multiplex 2	26.5	29.5	30.8	32.7	36.5	Neg
*mecA*	Monoplex	27.0	31.0	34.5	32.5	37.6	Neg
	Multiplex 2	27.2	31.2	33.9	32.5	35.9	Neg
*S. aureus*	Monoplex	27.0	30.5	31.8	34.0	38.7	Neg
	Multiplex 3	26.5	30.0	31.2	33.8	36.8	Neg
*E. faecalis*	Monoplex	29.6	33.0	34.1	36.3	37.4	Neg
	Multiplex 3	28.7	32.0	33.1	35.6	37.0	Neg
*P. aeruginosa*	Monoplex	27.9	30.8	30.9	35.3	38.1	Neg
	Multiplex 3	26.9	29.7	30.1	33.8	36.2	Neg
*C. krusei*	Monoplex	29.5	33.6	33.0	Neg	Neg	Neg
	Multiplex 3	26.8	30.2	31.7	32.2	Neg	Neg
*S. pneumoniae*	Monoplex	29.0	33.0	33.5	35.9	40	Neg
	Multiplex 3	28.7	32.5	33.0	34.6	37.2	Neg
*Staphylococcus* genus	Monoplex	29.3	33.4	33.9	36.4	40	Neg
	Multiplex 4	28.7	32.2	33.1	34.0	Neg	Neg
Enterococcus genus	Monoplex	29.8	33.1	34.1	35.6	36.2	Neg
	Multiplex 4	28.9	32.5	33.5	34.8	35.9	Neg
*Klebsiella* species	Monoplex	28.0	31.4	32.3	34.8	40	Neg
	Multiplex 4	27.5	30.7	31.7	33.9	36.8	Neg
*C. albicans*	Monoplex	28.6	31.6	33.1	34.8	Neg	Neg
	Multiplex 4	27.5	30.6	31.8	33.5	Neg	Neg

Results are presented as Ct-values obtained in one representative experiment. For each PCR, at least 2 independent spiking experiments were performed. The *Aspergillus* and CTX-M-1,9 PCR were not evaluated in this format as *Aspergillus* cultures could not reliably be quantified and the CTX-M-1,9 PCR was not evaluated in the clinical samples. The sensitivities of these PCRs were evaluated using DNA from pure cultures.

*Neg* no amplification detected

^a^Healthy human donor blood was spiked with different concentrations of live pathogens and processed by the Polaris method to yield the indicated amount of DNA (calculated as number of genomes) per PCR

### Clinical sample selection

For BSI-PCR validation, a total of 5570 blood cultures were obtained from 1679 ICU patients, including 732 (13%) specimens yielding growth of one or more microorganisms. Following eligibility criteria outlined above, we first selected samples paired with a BC being positive for any of the targets included in the BSI-PCR. Overall, this resulted in 347 samples (yielding 356 isolates) for validation (Table 4). Similarly, among the available 4838 culture-negative blood specimens, a selection of 200 samples was made. There were no BCs yielding growth of *A. baumannii, Candida (C.) krusei*, *C. tropicalis* or *Aspergillus* species. Therefore, specific PCRs addressing these targets were assessed analytically during BSI-PCR development, but could not be further validated. None of the clinical samples had to be excluded from analysis due to PCR inhibition (as indicated by the performance of the internal amplification control PCR).

**Table 4:** Sensitivity and Specificity of BSI-PCR per pathogen or target in clinical samples

Pathogen or target	Sensitivity			Specificity		
	BSI-PCR positive / BC positive	% (95%-CI)		BSI-PCR positive in 200 negative BC ^a^	% (95%-CI)	
A. Species-specific PCRs						
*E. faecalis* ^b^	20/27	74%	(54–89)	5	98%	(95–100)
*E. faecium* ^b^	33/50	66%	(51–79)	10	95%	(91–98)
*S. aureus*	28/35	80%	(63–92)	22	89%	(84–93)
*S. pneumoniae*	2/2	100%	(16–100)	5	98%	(94–99)
*A. baumannii*	0/0	NA		3	99%	(97–100)
*E. coli*	17/19	89%	(67–99)	15	93%	(88–96)
*Klebsiella* species	11/17	65%	(38–86)	1	100%	(97–100)
*P. aeruginosa*	27/29	93%	(77–99)	22	89%	(84–93)
*C. albicans*	0/27	0%	(0–13)	0	100%	(98–100)
*C. glabrata*	1/14	7%	(0–34)	0	100%	(98–100)
*C. krusei*	0/0	NA		1	100%	(97–100)
B. Generic PCRs						
*Enterococcus* genus ^b^	44/77	57%	(45–68)	4	98%	(95–99)
*Staphylococcus* genus ^b^	41/85	48%	(37–59)	12	94%	(90–97)
Coagulase negative *Staphylococi* ^b, c^	22/50	44%	(30–59)	12	94%	(90–97)
Gram-positive bacteria ^d^	51/196	26%	(20–33)	3	99%	(96–100)
Gram-negative bacteria ^d^	37/115	32%	(24–42)	5	98%	(94–100)
3*Candida*	7/31	23%	(10–41)	0	100%	(98–100)
Pan-*Aspergillus*	0/0	NA		2	99%	(96–100)

*BC* blood culture, *CI* confidence interval, *NA* no blood culture isolates available for evaluation

^a^Each PCR was evaluated in the same set of 200 culture-negative samples

^b^Selection criteria were applied to reduce the chance of blood culture being discordant positive by contamination

^c^Inferred from positivity in the *mecA* and/or *Staphylococcus* genus PCR and negativity in the *S. aureus* PCR

^d^For Gram-positive 8 pathogens were intrinsically not detectable by chosen primers and probes, excluding these resulted into a sensitivity of 27%. For Gram-negative 19 *Bacteroides* species were intrinsically not detectable, excluding these resulted into a sensitivity of 38%.

### Sensitivity of BSI-PCR

The sensitivity of individual PCRs within the multiplex setting was highly variable (Table 4). For bacterial pathogens, the observed detection rates ranged from 65 to 100% for PCRs targeting bacteria at the species level, and from 26 to 57% for PCRs targeting bacteria at their genus or Gram stain level. Sensitivity was 0 to 7% for PCRs targeting individual yeasts, and 23% for the 3Candida PCR targeting three species combined. Overall, BSI-PCR identified 138 (77%) of the 179 bacterial isolates that theoretically should be detected on species level, yet only 1 of the 41 fungal species. Of the residual 217 undetected isolates, 136 negative results were explained by the targeted pathogens not being intrinsically detectable by species-specific PCRs and 81 undetected pathogens were due to failure of PCRs. However, 54 (25%) of these isolates were correctly identified on genus level and/or molecular Gram stain. Sensitivity of BSI-PCR was similar for the 39 isolates cultured from 30 blood samples yielding a polymicrobial result, compared to the 317 isolates cultured from samples yielding only a single microorganism (41 versus 52%, *p* = 0.21).

### Specificity of BSI-PCR

Specificity of individual PCRs ranged from 89 to 100% in BC-negative samples (Table 4). However, given the multiplex setting combining 18 distinct tests, BSI-PCR was considered (completely) true negative in only 121 (60%) of 200 BC-negative samples. Thus, discordant positive PCR results were found among 79 culture-negative samples, yielding a total of 84 species-specific and 26 generic PCR results. Furthermore, BSI-PCR identified 90 pathogens that were non-congruent with BC growth in the 218 culture-positive samples selected for evaluation of species-specific PCRs. All together there were thus 174 discordant positive species-specific PCR results occurring among 4598 individual PCRs performed, yielding an overall specificity of 96%. These detections mainly concerned *S. aureus* (*n* = 43), *E. coli* (*n* = 38), and *P. aeruginosa* (*n* = 34; Table 5).

**Table 5:** Specifies-specific PCR detections in relation to blood culture results

Species-specific PCR	Congruent detection by BSI-PCR of 227 BC isolates		Additional detected pathogens by BSI-PCR	
	*n*	Ct-value	*n*	Ct-value
*E. faecalis*	21	32.1 (29.6-36.1)	21	37.2 (36.6-38.8)
*E. faecium*	35	36.1 (33.9-37.2)	21	39.1 (36.6-40.0)
*S. aureus*	28	34.9 (32.9-37.0)	43	40.0 (39.2-40.0)
*S. pneumonia*	2	33.9 (27.7-40.0)	7	39.6 (36.8-40.0)
*A. baumannii*	0		4	33.6 (32.7-33.9)
*E. coli*	17	34.0 (30.4-34.6)	38	36.6 (35.9-37.4)
*Klebsiella* species	11	31.5 (27.7-33.8)	1	34.1
*P. aeruginosa*	27	34.6 (31.8-37.1)	34	40.0 (38.4-40.0)
*C. albicans*	0	-	0	-
*C. glabrata*	1	28.3	0	-
*C. krusei*	0	-	5	36.0 (34.5-40.0)
Total Positive	142 (63%)	34.4 (31.5-36.7) ^a^	174 (4%) ^b^	38.6 (36.3-40.0)

Results incorporate all detections of species-specific PCRS of BSI-PCR and blood culture (*BC*) in 218 BC-positive (with 227 BC isolates) and 200 BC-negative samples, thus no selection criteria were applied for common BC contaminants. Each sample was evaluated for 11 species-specific PCRs thus contributing 11 results. Data are presented as frequencies, Ct-values as median (interquartile range)

^a^Ct-values were significantly lower in true positive than in discordant positive results (*p*<0.001)

^b^BSI-PCR identified 174 (4%) additional pathogens in 4371 PCRs evaluating pathogens not detected by BCs. Ct- values of 84 detected pathogens by BSI-PCR were significantly higher in samples with complete negative BC results than for detections in samples with positive BC (median 39.7 (IQR 37.0-40.0) versus Ct 37.4 (IQR 36.0-40.0), *p*=0.01)

### Impact of bacterial load on PCR performance

**Fig. 1:**
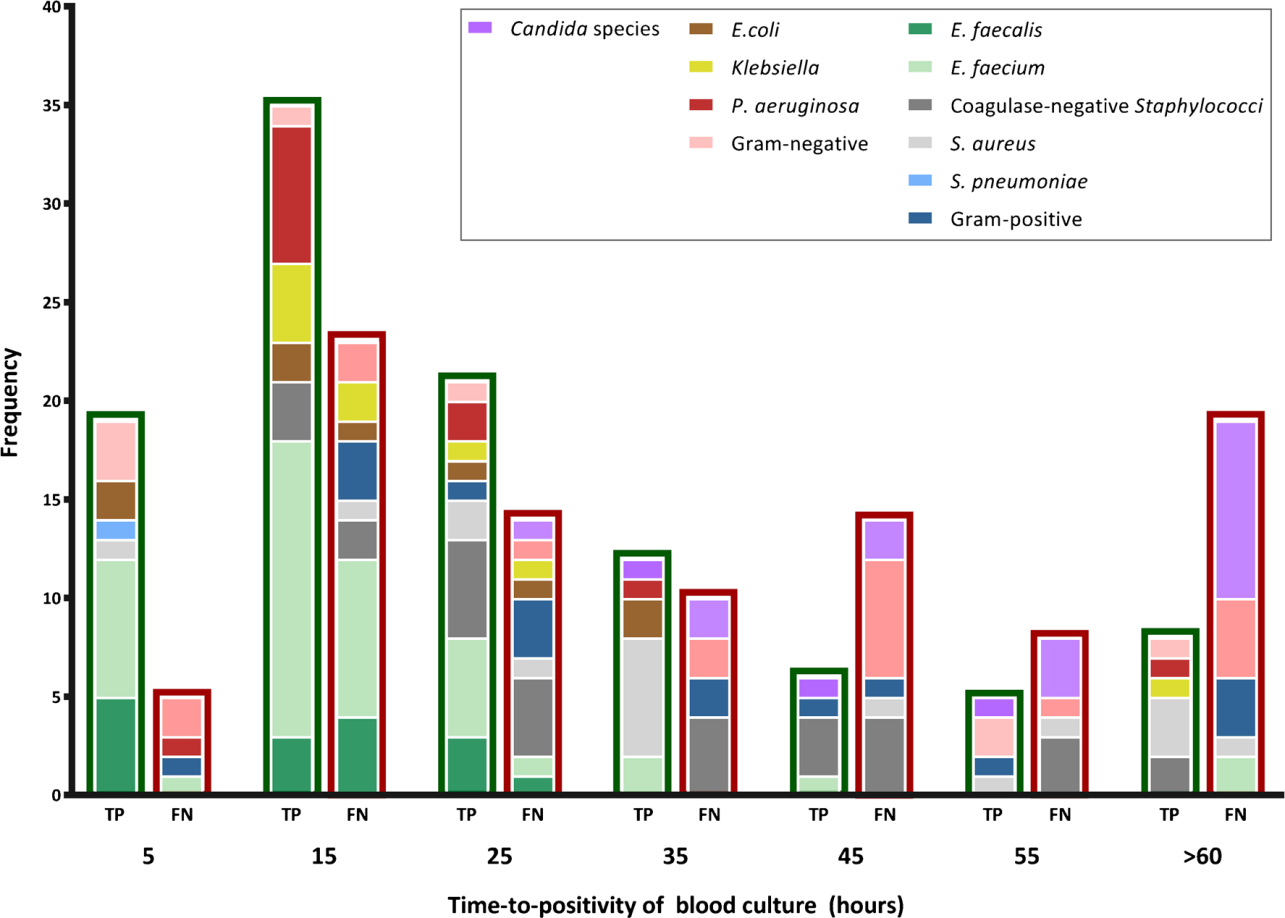
Time-to-positivity of blood culture in samples paired to true-positive versus false-negative PCR results. Frequency distributions of time-to-positivity are shown for 106 positive blood cultures associated with correctly detected pathogens by BSI-PCR (true positive (*TP*), bars with green border) and for 91 blood cultures with no congruent detection by BSI-PCR (false negative (*FN*), bars with red border)

To assess whether BSI-PCR sensitivity was related to pathogen load, we correlated time-to-positivity (TTP, a proxy of viable pathogen load in blood) for a subset of 197 BC-positive samples to the corresponding PCR results. The median TTP was 19.6 h (interquartile range (IQR) 11.2–32.3) for samples yielding a true positive PCR result and 35.2 h (IQR 16.3–54.1) for samples yielding a false negative result (Fig. 1). However, this difference may be partly explained by an overall reduced sensitivity of BSI-PCR for the detection of CNS and *Candida* species, the latter being known to be slow growers in conventional BC [34]. Ct-values of species-specific PCRs were significantly lower (indicating larger amounts of microbial DNA) in true positive compared to discordant positive test results (median Ct 34.4 (IQR 31.5–36.7) versus 38.6 (IQR 36.3–40.0), *p* < 0.001; Table 5). However, there was no overall correlation between Ct-value and TTP in a subset of 106 true positive results (Spearman correlation coefficient 0.15, *p* = 0.12), although for individual species Ct-values seemed to be higher at longer TTP except for *Staphylococci* (Fig. S1).

### Post hoc analyses of discrepant samples

Detection of *Candida* species in the clinical samples appeared especially poor. To exclude that underperformance of *Candida* PCRs in clinical samples was related to the multiplex set-up of BSI-PCR, we re-tested isolated DNA of 37 samples of the 45 *Candida-*positive BC in a simpler format (including only primers and probes for *Aspergillus* and *Candida* species). This resulted in an improved sensitivity for all *Candida* PCRs, yielding detection rates for *C. albicans* and *C. glabrata* of 41 and 45% at their species level, respectively. Additionally, all four samples with *C. parapsilosis* were now positive in the 3Candida PCR.

Among all pathogens evaluated, 82 bacterial isolates were not covered by any of the species- or genus-specific PCRs included in the test. However, 56 of these could potentially have been detected by the generic Gram stain PCR. Indeed, this was the case for 3 (12%) out of 26 Gram-positive and 12 (40%) out of 30 Gram-negative pathogens. All remaining 26 non-detected isolates (including *Bacteroides*, *Proteus*, *Micrococcus*, *Eggerthella*, and *Propionibacterium* species) contain mismatches in primer/probe regions of the 16S rDNA region targeted by the Gram stain PCRs, which prevented amplification.

Another portion of failed PCR detection could be due to unknown variations in the DNA sequence of individual pathogen strains, which may cause mismatch of primers and probes. In our study, this could have been the case for 13 samples containing pathogens that were detected by a generic PCR, but not by their corresponding species-specific PCR. Eight of these BC isolates were retrieved from the microbiology lab, DNA was extracted from pure culture, and the samples were then re-tested in a monoplex species-specific PCR. This resulted in correct pathogen identification in six (75%) of these samples, excluding DNA sequence variation as a cause of these detection failures.

### Limitations of the reference test

We performed three additional analyses to test the robustness of our estimations of BSI-PCR sensitivity. First, we assessed contamination of BC during blood sampling as a possible cause of false negative PCR results. For this purpose, we applied extra criteria for the selection of samples used to determine the sensitivity of the PCRs for Gram-positive bacteria (which are known to be frequent contaminants of BC). This resulted in the exclusion of 70 samples from our primary analysis. As a consequence, detection rates for *E. faecalis* (79%) and CNS (52%) improved, but not for other pathogens (Table S2). Second, we assessed the potential impact of concomitant antimicrobial drug use on the observed specificity of BSI-PCR. These drugs inhibit growth of viable pathogens present in BC and may thus be at the cause of an apparently discrepant PCR result. However, observed discordant positive rates were 42% for samples collected during antibacterial or antifungal treatment versus 38% for remaining samples (*p* = 0.51), making such effect unlikely. Finally, we assessed whether discrepant positive results of BSI-PCR related to other culture results observed within 72 h of the index BC sample (for cultures obtained in the ICU and patients treated in one of the study centers only). Overall, 23 (13%) of the 181 false-positive PCR-results in the 130 blood samples included in this analysis were in concordance to BC findings at a different time-point, and 66 (36%) results concurred with at least one culture obtained in the ICU from various other body sites or specimens (Table S3).

## Discussion

We designed a novel multiplex PCR assay for rapid pathogen identification directly in blood and assessed its diagnostic performance by comparing to BC results obtained from critically ill patients suspected of infection. Observed sensitivity of BSI-PCR was relatively high for the majority of individual bacterial pathogens, yet insufficient for yeasts and bacteria groups. However, *Candida* detection rates improved when PCRs were performed using a simplified set-up. Specificity was acceptable (i.e., > 89%) when PCRs were analyzed individually, but for the multiplex BSI-PCR taken as whole, discordant positive results were frequently observed due to the large number individual tests performed on each sample. Of note, all microbes that were additionally identified by BSI-PCR in our study were regarded as spurious (i.e., false positive) findings, yet in fact may have some clinical relevance. This is due to imperfect sensitivity of BC [2], which was used as the reference standard. Furthermore, PCR inhibition was never observed during the analysis of a large number of clinical blood samples, demonstrating that the Polaris method is a robust procedure for the preparation of human blood for multiplex PCR not only in case of healthy human blood samples [28], but also for critically ill patient samples.

The development of PCR assays for pathogen detection directly in whole blood is technically challenging. This results from low concentrations of circulating microbial DNA (related to usual pathogen loads of 1 to 10 CFU/mL), as well as high risk for inhibition of the PCR reaction due to an abundance of human DNA and substances like hemoglobin [35, 36]. As a consequence, PCR sensitivity is highly influenced by input sample volume and the method used for enrichment of microbial DNA. In comparison to the 40 mL blood commonly drawn during routine BC, molecular assays have a limited input volume (typically 1–10 mL), of which only a fraction ends up in each individual PCR. The combined Polaris and BSI-PCR method enables analysis of whole blood samples of 5 mL, yielding an equivalent of 0.71 mL blood input volume per PCR. This is considerably higher than most other molecular BSI assays [36]. In our study, we could not evaluate the effect of varying sample volume, but we did assess the effect of bacterial load on sensitivity. Ct-values were lower for true-positive than for false-positive PCR results, although we observed only a weak association between BSI-PCR pathogen detection rates and viable bacterial load in BC, deduced from TTP. This is most likely explained by the fact that TTP varies intrinsically among pathogen.

Besides targeting high input volumes, we aimed to improve PCR sensitivity by selecting multi-copy PCR targets on the microbial genome whenever possible (Table 1). The benefit of doing so is reflected by the observed difference between Ct-values obtained for *E. faecium* detections using 1 PCR target per genome (median Ct 36.1) and *E. faecalis* detections using 3–4 targets per genome (median Ct 32.1; Table 5). Of note, CNS detection was not based on multi-copy targets for a single PCR, but on positivity of either the MecA or the Staphylococcus genus PCR (with negativity on the *S. aureus* PCR). Simultaneous positivity on both PCRs was observed only for samples with relatively higher CNS loads (i.e., low MecA Ct-values; median 36.3 (IQR 35.6–38.1) versus 33.2 (IQR 32.1–34.3)). Apparently, the *Staphylococcus* genus PCR is less sensitive than the MecA PCR for the detection of low bacterial loads. This could be an advantage, since low CNS loads may be of little clinical consequence and are frequently caused by a contaminated blood draw.

Unfortunately, the detection of *Candida* species by BSI-PCR in its initial multiplex set-up was poor. In fact, higher sensitivities for fungal detection have been previously reported for other molecular BSI assays, including the LSF and T2Candida tests [8, 37]. This may be related to differences in the methodology used to isolate DNA from Candida cells. Both the LSF and T2Candida tests make use of a mechanical lysis process, whereas BSI-PCR uses chemical methods. Furthermore, in a trial evaluating the T2Candida assay, the majority of samples tested were spiked with laboratory grown *Candida* species rather than blood specimens obtained from patients with true Candidemia [37]. This may have impacted test performance as well. Indeed, during initial BSI-PCR development, *Candida* detection was deemed satisfactory in spiking experiments using laboratory-grown organisms. We therefore hypothesize that *Candida* isolates derived from clinical blood samples might be more difficult to process due to, for example, changes in cell wall characteristics [38]. On a similar note, we also observed inadequate detection by generic PCRs targeting bacteria at their genus level as well as by the molecular Gram stain. During early development of BSI-PCR, specificity of the generic molecular Gram-stain PCRs was increased by lowering cut-off Ct-values, even though this inherently lowered their sensitivity. We opted for this trade off, because of the increased risk of spuriously detecting blood contamination (which typically involves low bacterial loads) when using such broad generic PCR-targets. Since these PCRs were primarily designed to broaden microbial coverage of the test, their sensitivity will have only limited consequence for its clinical utility overall. These generic PCRs are mainly relevant for the minority of BSI cases where the causative pathogen is not covered by species-specific PCRs [15, 16].

Evaluation of novel diagnostic methods may be hampered by lack of a robust reference test [39]. This also applies to BSI-PCR, as its comparator (BC) has imperfect sensitivity in a setting of concurrent antimicrobial drug use [2]. Molecular tests are in theory less affected by this, because they are growth independent. Yet, we observed no effect of antimicrobial treatment on the rate of discordant positive BSI-PCR results. However, in fact, current knowledge about the kinetics of microbial DNA clearance upon initiation of antimicrobial treatment is very limited and in some cases this may be very fast [40]. So the assumption of the sensitivity of PCRs being robust during antimicrobial treatment may possibly be false. Of note, the Polaris method was designed to isolate DNA from intact pathogens only (dead or alive), but not cell-free DNA in plasma. Another consequence of the high sensitivity of PCRs also is an increased risk of discordant positive results due to contamination from the environment. Future improvement in producing DNA-free reagents may improve the utility of such general bacterial PCRs as the molecular Gram stain.

As this was the first evaluation of BSI-PCR, we deliberately focused on test sensitivity rather than on a clinical interpretation of discrepant positive results. For this purpose, we evaluated detection of a broad spectrum of microbes, while making use of clinical blood samples. However, because we did not enroll consecutive patients within a specific clinical domain, our study cannot provide estimations of positive and negative predictive values of BSI-PCR. As a consequence, test performance metrics (such as sensitivity and specificity) of BSI-PCR cannot be directly compared with those published for other molecular assays. In addition, we classified all pathogens detected by BSI-PCR but not by BC as “discordant positive” spurious findings, whereas in a clinical setting these might—in fact—be of relevance as illustrated by the concordance with other culture results observed in our explorative analysis. Thus, future studies will be necessary to further evaluate BSI-PCR performance and determine the clinical relevance of BSI-PCR discordant positive results within their full clinical context.In conclusion, sensitivity of BSI-PCR was promising for the majority of bacterial species. However, a better understanding of discordant positive results as well as an improvement of detection rates for PCRs targeting yeasts and bacterial groups are required before BSI-PCR can be commercialized as a robust tool for pathogen identification in ICU patients. Refinement of test sensitivity is currently focused on the use of larger blood input volumes and implementation of the BSI-PCR assay on a fully automated cartridge-base sample preparation and analysis platform. We thus expect BSI-PCR to become available as a point-of-care test, requiring only very little hands-on manipulation and yielding overall turnaround times of approximately 3 h.

## Electronic supplementary material

ESM 1 (PDF 466 kb)

## References

[1] Luyt CE, Brechot N, Trouillet JL, Chastre J (2014). Antibiotic stewardship in the intensive care unit. Crit Care.

[2] Afshari A, Schrenzel J, Ieven M, Harbarth S (2012). Bench-to-bedside review: rapid molecular diagnostics for bloodstream infection—a new frontier?. Crit Care.

[3] Perez KK, Olsen RJ, Musick WL, Cernoch PL, Davis JR, Land GA, Peterson LE, Musser JM (2013). Integrating rapid pathogen identification and antimicrobial stewardship significantly decreases hospital costs. Arch Pathol Lab Med.

[4] Ferrer R, Martin-Loeches I, Phillips G, Osborn TM, Townsend S, Dellinger RP, Artigas A, Schorr C, Levy MM (2014). Empiric antibiotic treatment reduces mortality in severe sepsis and septic shock from the first hour: results from a guideline-based performance improvement program. Crit Care Med.

[5] Garnacho-Montero J, Gutierrez-Pizarraya A, Escoresca-Ortega A, Fernandez-Delgado E, Lopez-Sanchez JM (2015). Adequate antibiotic therapy prior to ICU admission in patients with severe sepsis and septic shock reduces hospital mortality. Crit Care.

[6] Murray PR, Masur H (2012). Current approaches to the diagnosis of bacterial and fungal bloodstream infections in the intensive care unit. Crit Care Med.

[7] Peters RP, van Agtmael MA, Danner SA, Savelkoul PH, Vandenbroucke-Grauls CM (2004). New developments in the diagnosis of bloodstream infections. Lancet Infect Dis.

[8] Chang SS, Hsieh WH, Liu TS, Lee SH, Wang CH, Chou HC, Yeo YH, Tseng CP, Lee CC (2013). Multiplex PCR system for rapid detection of pathogens in patients with presumed sepsis—a systemic review and meta-analysis. PLoS One.

[9] Dark P, Blackwood B, Gates S, McAuley D, Perkins GD, McMullan R, Wilson C, Graham D, Timms K, Warhurst G (2015). Accuracy of LightCycler((R)) SeptiFast for the detection and identification of pathogens in the blood of patients with suspected sepsis: a systematic review and meta-analysis. Intensive Care Med.

[10] Tziolos N, Giamarellos-Bourboulis EJ (2016). Contemporary approaches to the rapid molecular diagnosis of sepsis. Expert Rev Mol Diagn.

[11] Chun K, Syndergaard C, Damas C, Trubey R, Mukindaraj A, Qian S, Jin X, Breslow S, Niemz A (2015). Sepsis pathogen identification. J Lab Autom.

[12] Mwaigwisya S, Assiri RA, O'Grady J (2015). Emerging commercial molecular tests for the diagnosis of bloodstream infection. Expert Rev Mol Diagn.

[13] Cambau E, Durand-Zaleski I, Bretagne S, Buisson CB, Cordonnier C, Duval X, Herwegh S, Pottecher J, Courcol R, Bastuji-Garin S, team Es (2017) Performance and economic evaluation of the molecular detection of pathogens for patients with severe infections: the EVAMICA open-label, cluster-randomised, interventional crossover trial. Intensive Care Med. 10.1007/s00134-017-4766-4 10.1007/s00134-017-4766-4 PMC5633620 28374097

[14] Warhurst G, Dunn G, Chadwick P, Blackwood B, McAuley D, Perkins GD, McMullan R, Gates S, Bentley A, Young D, Carlson GL, Dark P (2015). Rapid detection of health-care-associated bloodstream infection in critical care using multipathogen real-time polymerase chain reaction technology: a diagnostic accuracy study and systematic review. Health Technol Assess.

[15] Laupland KB, Church DL (2014). Population-based epidemiology and microbiology of community-onset bloodstream infections. Clin Microbiol Rev.

[16] Laupland KB, Zygun DA, Davies HD, Church DL, Louie TJ, Doig CJ (2002). Population-based assessment of intensive care unit-acquired bloodstream infections in adults: incidence, risk factors, and associated mortality rate. Crit Care Med.

[17] Landlinger C, Preuner S, Willinger B, Haberpursch B, Racil Z, Mayer J, Lion T (2009). Species-specific identification of a wide range of clinically relevant fungal pathogens by use of Luminex xMAP technology. J Clin Microbiol.

[18] Roschanski N, Fischer J, Guerra B, Roesler U (2014). Development of a multiplex real-time PCR for the rapid detection of the predominant beta-lactamase genes CTX-M, SHV, TEM and CIT-type AmpCs in Enterobacteriaceae. PLoS One.

[19] Yang S, Ramachandran P, Hardick A, Hsieh YH, Quianzon C, Kuroki M, Hardick J, Kecojevic A, Abeygunawardena A, Zenilman J, Melendez J, Doshi V, Gaydos C, Rothman RE (2008). Rapid PCR-based diagnosis of septic arthritis by early Gram-type classification and pathogen identification. J Clin Microbiol.

[20] van den Brand M, Peters RP, Catsburg A, Rubenjan A, Broeke FJ, van den Dungen FA, van Weissenbruch MM, van Furth AM, Koressaar T, Remm M, Savelkoul PH, Bos MP (2014). Development of a multiplex real-time PCR assay for the rapid diagnosis of neonatal late onset sepsis. J Microbiol Methods.

[21] Bode LG, van Wunnik P, Vaessen N, Savelkoul PH, Smeets LC (2012). Rapid detection of methicillin-resistant Staphylococcus aureus in screening samples by relative quantification between the mecA gene and the SA442 gene. J Microbiol Methods.

[22] Salehi E, Hedayati MT, Zoll J, Rafati H, Ghasemi M, Doroudinia A, Abastabar M, Tolooe A, Snelders E, van der Lee HA, Rijs AJ, Verweij PE, Seyedmousavi S, Melchers WJ (2016). Discrimination of aspergillosis, Mucormycosis, Fusariosis, and Scedosporiosis in formalin-fixed paraffin-embedded tissue specimens by use of multiple real-time quantitative PCR assays. J Clin Microbiol.

[23] Habets MN, Cremers AJ, Bos MP, Savelkoul P, Eleveld MJ, Meis JF, Hermans PW, Melchers WJ, de Jonge MI, Diavatopoulos DA (2016). A novel quantitative PCR assay for the detection of Streptococcus pneumoniae using the competence regulator gene target comX. J Med Microbiol.

[24] Grasman ME, Pettersson AM, Catsburg A, Koek AG, van Bodegraven AA, Savelkoul PH (2015). Tropheryma whipplei, a potential commensal detected in individuals undergoing routine colonoscopy. J Clin Microbiol.

[25] Koressaar T, Joers K, Remm M (2009). Automatic identification of species-specific repetitive DNA sequences and their utilization for detecting microbial organisms. Bioinformatics.

[26] Fouquier d'Herouel A, Wessner F, Halpern D, Ly-Vu J, Kennedy SP, Serror P, Aurell E, Repoila F (2011). A simple and efficient method to search for selected primary transcripts: non-coding and antisense RNAs in the human pathogen Enterococcus faecalis. Nucleic Acids Res.

[27] Phillippy AM, Ayanbule K, Edwards NJ, Salzberg SL (2009). Insignia: a DNA signature search web server for diagnostic assay development. Nucleic Acids Res.

[28] Loonen AJ, Bos MP, van Meerbergen B, Neerken S, Catsburg A, Dobbelaer I, Penterman R, Maertens G, van de Wiel P, Savelkoul P, van den Brule AJ (2013). Comparison of pathogen DNA isolation methods from large volumes of whole blood to improve molecular diagnosis of bloodstream infections. PLoS One.

[29] Klein Klouwenberg PM, Ong DS, Bos LD, de Beer FM, van Hooijdonk RT, Huson MA, Straat M, van Vught LA, Wieske L, Horn J, Schultz MJ, van der Poll T, Bonten MJ, Cremer OL (2013). Interobserver agreement of Centers for Disease Control and Prevention criteria for classifying infections in critically ill patients. Crit Care Med.

[30] Hall KK, Lyman JA (2006). Updated review of blood culture contamination. Clin Microbiol Rev.

[31] den Heijer CD, van Bijnen EM, Paget WJ, Pringle M, Goossens H, Bruggeman CA, Schellevis FG, Stobberingh EE, Team AS (2013). Prevalence and resistance of commensal Staphylococcus aureus, including meticillin-resistant S aureus, in nine European countries: a cross-sectional study. Lancet Infect Dis.

[32] Rottier WC, Bamberg YR, Dorigo-Zetsma JW, van der Linden PD, Ammerlaan HS, Bonten MJ (2015). Predictive value of prior colonization and antibiotic use for third-generation cephalosporin-resistant enterobacteriaceae bacteremia in patients with sepsis. Clin Infect Dis.

[33] Gruber K (2015). Here, there, and everywhere: from PCRs to next-generation sequencing technologies and sequence databases, DNA contaminants creep in from the most unlikely places. EMBO Rep.

[34] Lai CC, Wang CY, Liu WL, Huang YT, Hsueh PR (2012). Time to positivity of blood cultures of different Candida species causing fungaemia. J Med Microbiol.

[35] Al-Soud WA, Radstrom P (2001). Purification and characterization of PCR-inhibitory components in blood cells. J Clin Microbiol.

[36] Opota O, Jaton K, Greub G (2015). Microbial diagnosis of bloodstream infection: towards molecular diagnosis directly from blood. Clin Microbiol Infect.

[37] Mylonakis E, Clancy CJ, Ostrosky-Zeichner L, Garey KW, Alangaden GJ, Vazquez JA, Groeger JS, Judson MA, Vinagre YM, Heard SO, Zervou FN, Zacharioudakis IM, Kontoyiannis DP, Pappas PG (2015). T2 magnetic resonance assay for the rapid diagnosis of candidemia in whole blood: a clinical trial. Clin Infect Dis.

[38] Hall RA (2015). Dressed to impress: impact of environmental adaptation on the Candida albicans cell wall. Mol Microbiol.

[39] Reitsma JB, Rutjes AW, Khan KS, Coomarasamy A, Bossuyt PM (2009). A review of solutions for diagnostic accuracy studies with an imperfect or missing reference standard. J Clin Epidemiol.

[40] Peters RP, van Agtmael MA, Gierveld S, Danner SA, Groeneveld AB, Vandenbroucke-Grauls CM, Savelkoul PH (2007). Quantitative detection of Staphylococcus aureus and Enterococcus faecalis DNA in blood to diagnose bacteremia in patients in the intensive care unit. J Clin Microbiol.

