# Supplementary material for: Development and first evaluation of a novel multiplex real-time PCR on whole blood samples for rapid pathogen identification in critically ill patients with sepsis
Source: Eur J Clin Microbiol Infect Dis. 2018 Apr 26;37(7):1333–44. doi: 10.1007/s10096-018-3255-1 (PMC6015113; doi:10.1007/s10096-018-3255-1)
Supplement: Supplementary file 1 — (PDF 466 kb) [file 10096_2018_3255_MOESM1_ESM.pdf]

SUPPLEMENTAL DIGITAL CONTENT

Development and first evaluation of a novel multiplex real-time PCR on whole blood samples for rapid pathogen identification in critically ill patients with sepsis

Kirsten van de Groep[1,2]*#, Martine P. Bos[3]*, Paul H.M. Savelkoul[3-5], Anna Rubenjan[3], Christel Gazenbeek[3], Willem J.G. Melchers[6], Tom van der Poll[7,8], Nicole P. Juffermans[9], David S.Y. Ong[2,10], Marc J.M. Bonten[1,10], Olaf L. Cremer[2], *on behalf of the MARS consortium*

[1]Department of Epidemiology, Julius Center for Health Sciences and Primary Care, University Medical Center Utrecht, Utrecht University, the Netherlands. [2]Department of Intensive Care Medicine, University Medical Center Utrecht, Utrecht University, the Netherlands. [3]Microbiome, Amsterdam, the Netherlands. [4]Department of Medical Microbiology & Infection Control, VU University Medical Center, Amsterdam, the Netherlands. [5]Department of Medical Microbiology, Maastricht University Medical Center, Maastricht, the Netherlands. [6]Department of Medical Microbiology, Radboud University Medical Center, Nijmegen, the Netherlands. [7]Center of Experimental and Molecular Medicine, Academic Medical Center, University of Amsterdam, the Netherlands. [8]Division of Infectious Diseases, Academic Medical Center, University of Amsterdam, the Netherlands. [9]Department of Intensive Care, Academic Medical Center, University of Amsterdam, the Netherlands. [10]Department of Medical Microbiology, University Medical Center Utrecht, Utrecht University, the Netherlands.

*KvdG and MPB contributed equally to this work

#Corresponding author: Kirsten van de Groep, MD. Email: k.vandegroep-3@umcutrecht.nl

Mailing address: Room F06.149, P.O. Box 85500, 3508 GA Utrecht, The Netherlands.

Telephone: +31 88 756 1124

Appendix I. Table S1. Strains used to evaluate coverage and specificity of the monoplex PCR assays

Appendix II. Table S2. Apparent detection rates of BSI-PCR for common blood culture contaminants

Appendix III. Table S3. Concordance between discordant positive BCI-PCR results and various (other) specimens sampled within a three-day time window from the index blood culture

Appendix IV. Figure S1. Ct-values of true positive BSI-PCR results and Time-to-positivity of paired blood cultures (*n*=106)

APPENDIX I

Table S1. Strains used to evaluate coverage and specificity of the monoplex PCR assays

**Species**	**Strain name / Source [a]**
Acinetobacter baumannii	ATCC 19606
Acinetobacter baumannii	RUH 875/LMG 10543
Acinetobacter baumannii	RUH 134/ LMG 10541
Acinetobacter baumannii	LUH 5875/ LMG 25784
Acinetobacter baumannii	clinical isolates (5)
Acinetobacter calcoaceticus	ATCC 23055
Acinetobacter genomo species 13TU	ATCC 17903
Acinetobacter genomo species 3	ATCC 19004
Acinetobacter haemolyticus	ATCC 17906
Acinetobacter johnsonii	ATCC 17909
Acinetobacter junii	ATCC 17908
Acinetobacter lwoffi	ATCC 15309
Actinomyces spp	clinical isolate (1)
Aerococcus viridans	clinical isolate (1)
Aspergillus candida	clinical isolate (1)
Aspergillus clavatus	clinical isolate (1)
Aspergillus flavus	clinical isolate (1)
Aspergillus fumigatus	clinical isolate (1)
Aspergillus nidulans	clinical isolate (1)
Aspergillus niger	clinical isolate (1)
Aspergillus terreus	clinical isolate (1)
Bacillius cereus	DSM4313
Bacteroides fragilis	ATCC 25282
Candida albicans	ATCC 90029
Candida albicans	ATCC 90030
Candida albicans	ATCC 90028
Candida albicans	clinical isolate (1)
Candida glabrata	ATCC 15545
Candida krusei	ATCC 6258
Candida krusei	clinical isolate (1)
Candida parapsilosis	ATCC 90018
Candida parapsilosis	ATCC 22019
Candida tropicalis	clinical isolates (2)
Citrobacter freundii	ATCC 8090
Citrobacter freundii	clinical isolate (1)
Citrobacter koseri	ATCC 27028
Clostridium bifermentans	ATCC 638
Corynebacterium xerosis	ATCC 373
Cronobacter sakazakii	clinical isolate (1)
Eikenella corrodens	DSM 8340
Enterobacter aerogenes	ATCC 13048
Enterobacter aerogenes	clinical isolate (1)
Enterobacter cloacae	ATCC 23373
Enterobacter cloacae	ATCC 13047
Enterobacter cloacae	clinical isolate (1)
Enterococcus avium	clinical isolate (1)

APPENDIX I. (continuation)

**Species**	**Source / Strain name** [a]
Enterococcus durans	clinical isolate (1)
Enterococcus faecalis	CDC NY 3
Enterococcus faecalis	ATCC 29212
Enterococcus faecalis	CDC NY 5
Enterococcus faecalis	clinical isolates (14)
Enterococcus faecium	ATCC 35667
Enterococcus faecium	CDC NY2
Enterococcus faecium	clinical isolates (12)
Enterococcus gallinarum	CDC NY4
Escherichia coli	ATCC 25922
Escherichia coli	ATCC 35218
Escherichia coli	ATCC 35323
Escherichia coli	ATCC 35150
Escherichia coli	ATCC 11775T
Escherichia coli	clinical isolates (11)
Escherichia coli (CTXM-1)	clinical isolates (2)
Escherichia coli (CTXM-9)	clinical isolates (3)
Escherichia vulneris	DSM 4564
Haemophilus influenza	ATCC 49247
Klebsiella oxytoca	ATCC 13182
Klebsiella oxytoca	DSM 7342
Klebsiella oxytoca	clinical isolates (5)
Klebsiella planticola	ATCC 33531
Klebsiella pneumoniae	ATCC BAA-1705
Klebsiella pneumoniae	ATCC 13884
Klebsiella pneumoniae	ATCC 13887
Klebsiella pneumoniae	ATCC 13883
Klebsiella pneumoniae	clinical isolates (7)
Klebsiella terrigena	ATCC 33257
Lactobacillus acidophilus	DSM 20079
Lactococcus lactis	DSM 20481
Listeria monocytogenes	ATCC 15313
Moraxella catarrhalis	clinical isolate (1)
Morganella morganii	clinical isolate (1)
Neisseria meningitidis	clinical isolate (1)
Propionibacterium acnes	DSM 16379
Proteus mirabilis	ATCC 29906
Pseudomonas aeruginosa	ATCC 27853
Pseudomonas aeruginosa	ATCC 10145T
Pseudomonas aeruginosa	clinical isolates (11)
Pseudomonas fluorescens	ATCC 13525
Pseudomonas putida	ATCC 12633
Salmonella enteritidis	ATCC 13076T
Salmonella enteritidis	clinical isolate (1)
Salmonella typhimurium	ATCC 14028
Salmonella typhimurium	clinical isolate (1)
Serratia marcescens	ATCC 13880

APPENDIX I. (continuation)

Species	Source / Strain name [a]
Serratia marcescens	DSM12481
Serratia marcescens	DSM17174
Serratia marcescens	clinical isolate (1)
Shigella flexneri	ATCC 12022
Staphylococcus aureus	ATCC 25923
Staphylococcus aureus	ATCC 29213
Staphylococcus aureus	ATCC 43300
Staphylococcus aureus	NCTC8325
Staphylococcus aureus	ATCC 12600T
Staphylococcus aureus	Newman
Staphylococcus aureus	clinical isolates (7)
Staphylococcus aureus	clinical isolates MRSA (3)
Staphylococcus capitis	LMD 89175
Staphylococcus capitis	clinical isolate (1)
Staphylococcus chromogenus	ATCC 43764
Staphylococcus epidermidis	ATCC 14990T
Staphylococcus epidermidis	ATCC 12228
Staphylococcus epidermidis	clinical isolates (2)
Staphylococcus haemolyticus	clinical isolates (2)
Staphylococcus hominis	clinical isolates (2)
Staphylococcus hyicus	NCTC 10350T
Staphylococcus hyicus	clinical isolate (1)
Staphylococcus saprophyticus	clinical isolates (3)
Staphylococcus warneri	clinical isolate (1)
Staphylococcus lugdunensis	clinical isolate (1)
Stenotrophomonas maltophilia	ATCC 13637
Stenotrophomonas maltophilia	clinical isolate (1)
Streptococcus agalactiae	ATCC 13813
Streptococcus agalactiae	clinical isolates (3)
Streptococcus mitis	ATCC 49456
Streptococcus mitis	clinical isolate (1)
Streptococcus oralis	ATCC 35037
Streptococcus parasanguinis	ATCC 15912
Streptococcus pneumoniae	TIGR4
Streptococcus pneumoniae	ATCC 49619
Streptococcus pneumoniae	ATCC 6305
Streptococcus pneumoniae	D39
Streptococcus pseudopneumoniae	ATCC BAA-960
Streptococcus pyogenes	ATCC 19615
Streptococcus sanguinis	ATCC 10556
Veillonella parvula	ATCC 10790
Yersinia enterocolitica	ATCC 23715
Yersinia enterocolitica	clinical isolates (2)

[a] Clinical isolates were retrieved from the department of Medical Microbiology and Infection Prevention, VU Medical Center, (Amsterdam, the Netherlands) except for the *Aspergillus* and *Candida* isolates which were retrieved from the department of Medical Microbiology, Radboud University Nijmegen Medical Centre, (Nijmegen, the Netherlands). The number of different clinical isolates evaluated is given in brackets.

APPENDIX II.

Table S2. Apparent detection rates of BSI-PCR for common blood culture contaminants

Pathogen	Primary analysis (n=159) [a]		Secondary analysis (n=89) [b]	
	Blood culture positive	BSI-PCR positive	Blood culture positive	BSI-PCR positive
E. faecium	50	33 (66)	36	23 (64)
E. faecalis	27	20 (74)	14	11 (79)
Coagulase negative Staphylococci (CNS)	50	22 (44)	25	13 (52)
Other Gram-positive [c]	32	3 (2)	14	0

Results are presented as frequencies (row percentages).

[a] Pathogens were classified as possible contaminant and excluded if ≥1 of the following criteria was fulfilled:
- Poly-microbial results from corresponding blood culture with Enterococci and CNS
- Negative blood culture or blood culture with different pathogen on same day

[b] Pathogens were classified as possible contaminant and excluded if ≥1 of the additional criteria was fulfilled:
- Poly-microbial results from corresponding BC with other Gram-positive pathogens (*n*=8)
- Negative BC within next two days (*n*=54)
- Sample was not drawn under a therapeutic antimicrobial course and no initiation of therapeutic antimicrobial in next two days (*n*=18)

[c] Excluding *S. aureus* and *S. pneumonia.*

APPENDIX III.

Table S3. Concordance between discordant positive BCI-PCR results and various (other) specimens sampled within a three-day time window from the index blood culture

Discordant-positive PCR result	(*n*)	Specimen or site					
		**Blood [a] (*n*=103)**	**Sputum (*n*=118)**	**Throat (*n*=123)**	**Rectum (*n*=125)**	**Body-surface [b] (*n*=78)**	**Deep/Sterile [c] (*n*=70)**
E. faecalis	(23)	2	0	0	0	1	7
E. faecium	(19)	6	0	0	0	1	7
S. aureus	(30)	1	4	4	4	6	3
S. pneumoniae	(5)	0	0	0	0	0	0
A. baumannii	(4)	1	1	0	0	0	0
E. coli	(34)	0	2	5	11	1	2
P. aeruginosa	(18)	0	1	1	1	0	0
Enterococcus genus	(13)	4	0	0	0	1	6
Staphylococcus genus	(17)	5	0	1	0	4	1
Gram-positive	(8)	4	0	0	0	4	3
Gram-negative	(7)	0	2	1	3	0	0
3*Candida*	(1)	0	0	0	0	0	0
Pan-*Aspergillus*	(2)	0	0	0	0	0	0
**Total (%)**	**(181)**	**23 (13)**	**10 (6)**	**12 (7)**	**19 (10)**	**18 (10)**	**29 (16)**

Results are presented as concordance rates between 181 discordant positive BSI-PCR results (occurring among 130 blood samples used in the primary analysis) and various other specimens obtained for culture during ICU-stay within ±3 days of the index sample. These included twice weekly surveillance samples of throat, sputum, and rectum collected as part of local SDD protocols that were in place in both study centers, as well as cultures obtained by order of the attending physician. Number of performed cultures varied for each evaluated BSI-PCR sample from 0-3 per evaluated site/specimen and one discordant positive PCR result can be in concordance with multiple sites or specimens.

[a] Concerns other blood cultures than the paired index blood culture. The latter was initially used to evaluate BSI-PCR result and was collected at exactly the same moment and from same catheter hub or venipuncture site as the BSI-PCR sample.
[b] Body-surface sites include, among others, skin or wound swabs, and catheter tip cultures.
[c] Deep/sterile specimens include bronchoalveolar lavage fluid, urine, ascites, drain fluid, liquor, pus, (post-mortem) tissue biopsies.

APPENDIX IV.

Figure S1. Ct-values of true positive BSI-PCR results and time-to-positivity of paired blood cultures (*n*=106)

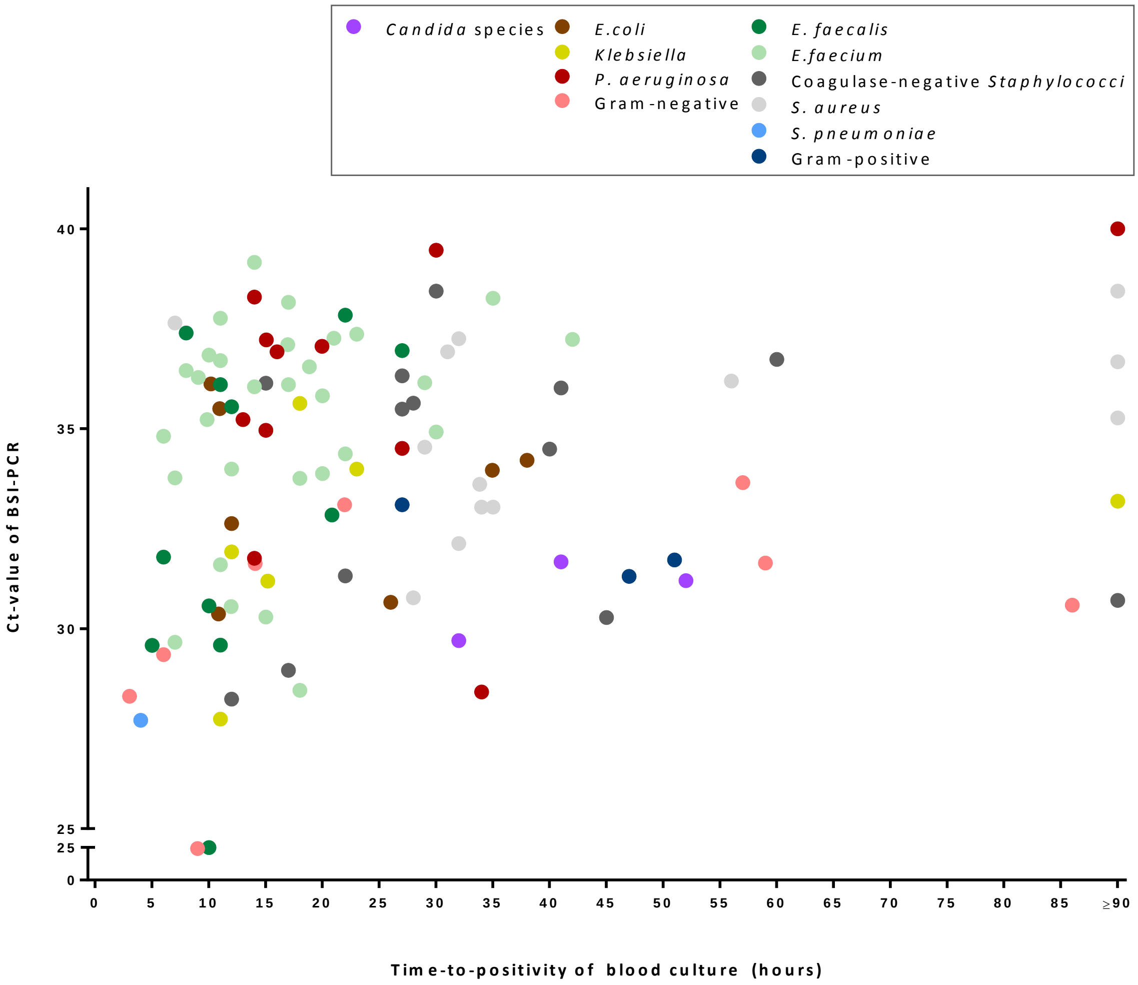

Data points show paired detections of pathogens by both blood culture and BSI-PCR, including identification of *E. faecium* (n=30), *S. aureus* (n=13), CNS (n=13), *E. faecalis* (n=11), *P. aeruginosa* (n=11), and other pathogens (n=28). There was no overall correlation between Ct-value and time-to-positivity: Spearman's rho=0.15, *p*=0.12.
